# Ethylene enhances root water transport and aquaporin expression in trembling aspen (*Populus tremuloides*) exposed to root hypoxia

**DOI:** 10.1186/s12870-021-02995-7

**Published:** 2021-05-21

**Authors:** Xiangfeng Tan, Mengmeng Liu, Ning Du, Janusz J. Zwiazek

**Affiliations:** 1 Department of Renewable Resources, University of Alberta, AB T6G 2E3 Edmonton, Canada; 2 Institute of Soil and Water Resources and Environmental Science, Zhejiang University, 310058 Hangzhou, China; 3 Institute of Ecology and Biodiversity, School of Life Science, Shandong University, 266237 Qingdao, China

**Keywords:** aquaporin, ethylene, flooding, oxygen, hydraulic conductance

## Abstract

**Background:**

Root hypoxia has detrimental effects on physiological processes and growth in most plants. The effects of hypoxia can be partly alleviated by ethylene. However, the tolerance mechanisms contributing to the ethylene-mediated hypoxia tolerance in plants remain poorly understood.

**Results:**

In this study, we examined the effects of root hypoxia and exogenous ethylene treatments on leaf gas exchange, root hydraulic conductance, and the expression levels of several aquaporins of the plasma membrane intrinsic protein group (PIP) in trembling aspen (*Populus tremuloides*) seedlings. Ethylene enhanced net photosynthetic rates, transpiration rates, and root hydraulic conductance in hypoxic plants. Of the two subgroups of PIPs (PIP1 and PIP2), the protein abundance of PIP2s and the transcript abundance of *PIP2*;*4* and *PIP2*;*5* were higher in ethylene-treated trembling aspen roots compared with non-treated roots under hypoxia. The increases in the expression levels of these aquaporins could potentially facilitate root water transport. The enhanced root water transport by ethylene was likely responsible for the increase in leaf gas exchange of the hypoxic plants.

**Conclusions:**

Exogenous ethylene enhanced root water transport and the expression levels of *PIP2*;*4* and *PIP2*;*5* in hypoxic roots of trembling aspen. The results suggest that ethylene facilitates the aquaporin-mediated water transport in plants exposed to root hypoxia.

**Supplementary Information:**

The online version contains supplementary material available at 10.1186/s12870-021-02995-7.

## Background

Plant roots require sufficient amount of O_2_ for respiration and growth [1]. Contrary to anoxia, that is characterized by a total absence of O_2_, plant roots commonly experience periods of hypoxia, especially when O_2_ availability is reduced due to waterlogging [2] or soil compaction [3, 4]. Even in a normoxic environment, O_2_ gradients exist across tissues due to the differences in metabolic rates [5]. Although most of the terrestrial plant species cannot survive long-term O_2_ deficiency, some plants have developed effective strategies to either increase O_2_ delivery to roots or to tolerate short-term disruptions of the metabolism and the resulting toxicity [6].

Root hypoxia commonly alters plant water relations and may cause wilting [7, 8]. This effect was attributed to changes in the root system architecture [9] and an inhibition of aquaporin-mediated root water transport resulting in a decrease of root hydraulic conductivity [10, 11]. Aquaporins play a central role in plant water relations by regulating water flow resistance, which is especially important for the radial pathway in roots [9]. Aquaporin-mediated and apoplastic water transport interact with each other and regulate plant water balance [7, 12]. In addition to water, aquaporins can transport other small molecules including CO_2_ [13], NH_3_ [14] and O_2_ [15]. Plant aquaporins show high diversity with more than 30 different homologs in all of the examined species [9]. In higher plants, five subfamilies of aquaporins have been described including the PIPs (plasma membrane intrinsic proteins), TIPs (tonoplast membrane intrinsic proteins), NIPs (nodulin 26-like intrinsic proteins), SIPs (small intrinsic proteins) and XIPs (uncategorized intrinsic proteins).

Aquaporins mediate root hydraulic adjustments in response to environmental factors [16]. Aquaporin expression [15], membrane trafficking [17], and posttranslational regulation [18] have been implicated in regulating cell-to-cell water transport in response to environmental stress. The closure of aquaporins through intracellular acidosis [11] and energy deprivation [19] was also proposed to be among the reasons for the decreased water uptake by hypoxic roots. Recently, a potassium-dependent O_2_ sensing pathway was proposed in *Arabidopsis*, which may regulate the aquaporin activity [20]. Although the inhibition of root hydraulic conductivity by hypoxia is a commonly observed phenomenon, it cannot be clearly correlated with the expression levels of water-transporting aquaporins and both decreases and increases of gene expression levels encoding for different PIP2 aquaporins have been reported in hypoxic roots [7, 21]. On the other hand, increased gene expression by root hypoxia of the NIP and PIP1 aquaporins was reported to be involved in the transport of lactate [22, 23], CO_2_ [24], and O_2_ [15] in plants, suggesting a possible role of these aquaporins in hypoxia tolerance.

The inhibitory effects of hypoxia on root water transport in some plants could be partly reversed by the exposure of roots to exogenous ethylene [19]. Ethylene synthesis and accumulation in plants can be induced by various biotic and abiotic stress, including drought [25], wounding [26], and flooding stresses [27], and is produced through the Yang cycle [28]. The effects of ethylene are mediatedby the downstream transcriptional activities of a group of Ethylene Response Factors (ERFs), which are rapidly upregulated following an exposure of plants to hypoxia [29]. Ethylene can improve plant hypoxia tolerance through the stimulation of adventitious roots and aerenchyma formation [29–31], however, only a few studies have focused on the relationship between ethylene and aquaporin-mediated water transport [31]. Aquaporins were reported to be involved in the ripening of grape berry and latex yield of rubber tree that were regulated by ethylene [32, 33] and it was recently reported that the cell water transport in *Arabidopsis* was increased by ethylene through the enhanced C-Terminal phosphorylation of *At*PIP2;1 [29]. While these findings provide possible links between ethylene and aquaporin abundance as well as posttranslational regulation of aquaporins, more information is still needed for comprehensive understanding of the processes involved in regulation of root water transport by ethylene.

In the present study, we examined the effects of root hypoxia and exogenous ethylene application on gas exchange, water transport and the expression of ERF and aquaporin genes in trembling aspen (*P. tremuloides*) seedlings to shed more light on the regulation of aquaporin-mediated water transport by O_2_ deficiency and ethylene. It was hypothesized that the effects of ethylene on root hydraulics under hypoxic conditions involve changes inthe aquaporin gene expression.

## Results

### Leaf gas exchange and light responses

**Fig. 1:**
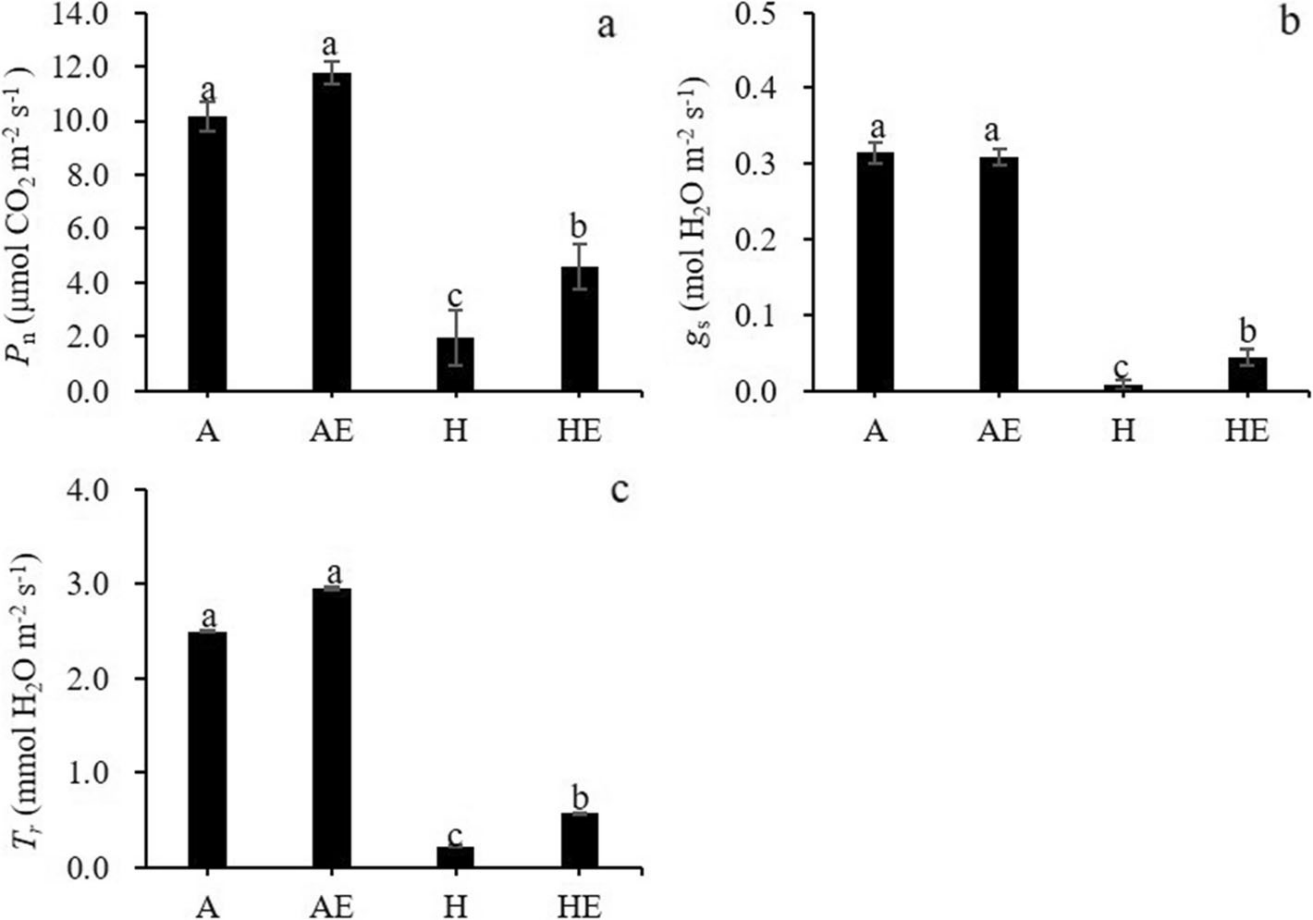
Net photosynthesis (*P*_n_, a), stomatal conductance (*g*_s_, b) and transpiration rates (*T*_r_, c) in well-aerated trembling aspen (*P. tremuloides*) plants (A), well-aerated plants treated with ethylene (AE), plants exposed to hypoxia (H) and subjected to hypoxia and ethylene treatment (HE) for one week. Means ± SE (*n* = 6) are shown. Different letters indicate significant difference (*P* ≤ 0.05)

Hypoxia treatments significantly reduced leaf net photosynthesis rate (*P*_n_), transpiration rate (*T*_r_) and stomatal conductance (*g*_s_) (Table S1; Fig. 1). Ethylene-treated plants showed significantly higher *P*_n_, *g*_s_ and *T*_r_ compared with plants without ethylene treatment under hypoxic conditions, but not under aerated conditions (Fig. 1).

To evaluate the effects of treatments on leaf gas exchange under varying light conditions, we determined the light responses of *P*_n_ and *g*_s_. Hypoxia treatments significantly decreased the light saturation point (*I*_m_) and the level of *P*_n_ at light saturation (*P*_m_) (Table S1; Fig. S1a). Ethylene treatments significantly increased *I*_m_ but not *P*_m_ in hypoxic plants (Table 1). When exposed to hypoxia, ethylene-treated plants showed significantly higher *I*_m_ compared with the plants that were not exposed to ethylene (Table S1). Light response of *g*_s_ showed that *g*_s_ gradually increased with increasing light intensity under aerated conditions, but light intensity had no effect on *g*_s_ under hypoxic conditions (Fig. S1b).

**Table 1:** Comparison of estimated light-saturated photosynthesis (*P*_m_) and light saturation point (*I*_m_) in trembling aspen (*P. tremuloides*) plants after one week of treatments. Means ± SE (*n* = 3) are shown. Different letters indicate significant difference (*P* ≤ 0.05)

	A	AE	H	HE
*P*_m_ (μmol CO_2_ m^-2^ s^-1^)	17.52 ± 0.93a	18.71 ± 1.02ab	3.65 ± 1.88c	9.83 ± 0.38bc
*I*_m_ (μmol m^-2^ s^-1^)	1481.16 ± 101.89a	1402.99 ± 43.43a	747.67 ± 163.74b	975.33 ± 115.59c

## Root hydraulic conductance

**Fig. 2:**
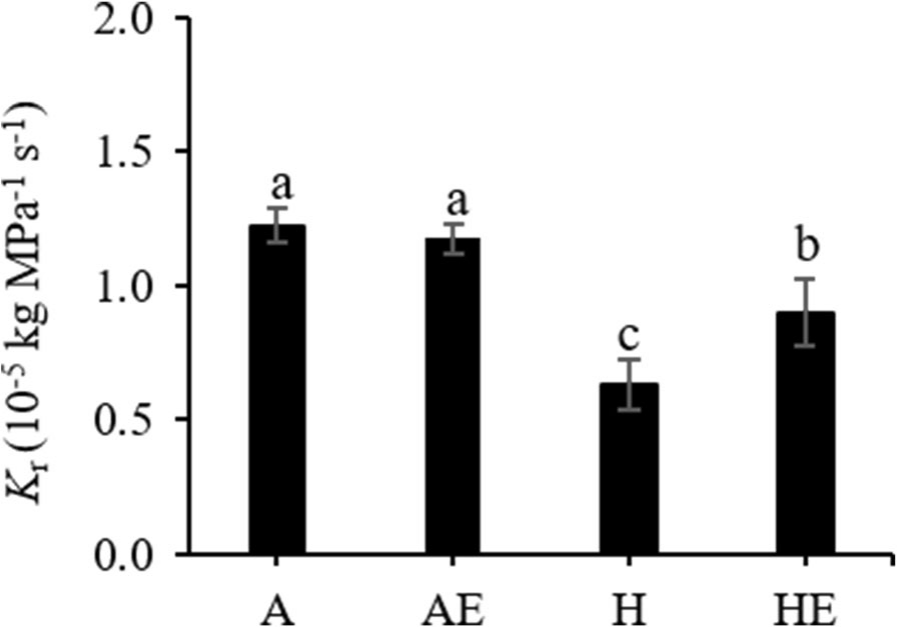
Root hydraulic conductance (*K*_r_) in well-aerated trembling aspen (*P. tremuloides*) plants (A), well-aerated plants treated with ethylene (AE), plants exposed to hypoxia (H) and subjected to hypoxia and ethylene treatment (HE) for one week. Means ± SE (*n* = 6) are shown. Different letters indicate significant difference (*P* ≤ 0.05)

Hypoxia resulted in a drastic decrease of root hydraulic conductance (*K*_r_) (Table S1; Fig. 2). Exogenous ethylene did not affect *K*_r_ in well-aerated plants, but in the plants subjected to hypoxia, ethylene treatment significantly increased *K*_r_ by about 20 % compared with the hypoxic plants that were not treated with ethylene (Fig. 2).

## mRNA expression profiling

**Fig. 3:**
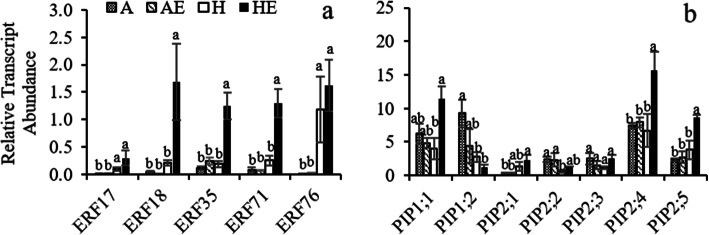
Transcript abundance of ethylene responsive factors (*ERF*s, a) and plasma membrane intrinsic proteins (*PIP*s, b) in well-aerated trembling aspen (*P. tremuloides*) plants (A), well-aerated plants treated with ethylene (AE), plants exposed to hypoxia (H) and subjected to hypoxia and ethylene treatment (HE) for one week. Means ± SE (*n* = 4–6) are shown. Different letters indicate significant difference compared between each gene (*P* ≤ 0.05)

After one week of treatments, mRNA expression profiling was carried out by qRT-PCR. Hypoxia treatments significantly affected the relative transcript abundance of the five ethylene responsive factors (*ERFs*) (Table S1). Significant effects of ethylene were detected in *ERF35* and *ERF71*, and interactions between hypoxia and ethylene treatments were also detected in these two genes (Table S1). Ethylene treatments significantly increased the relative transcript abundance of *ERF18, ERF35* and *ERF71* under hypoxic conditions, but not under aerated conditions (Fig. 3a). In contrast, the relative transcript abundance of the *ERF17* and *ERF76* in hypoxic plants were significantly higher compared with well-aerated plants, but showed no change compared with the plants exposed to both hypoxia and ethylene treatments (Fig. 3a).

Considering *PIP*s expression level, hypoxia significantly decreased the relative transcript abundance of *PIP1*;*2* and *PIP2*;*2*, but significantly increased the relative transcript abundance of *PIP2*;*1* and *PIP2*;*5* (Table S1; Fig. 3b). Ethylene-treated plants exhibited significantly higher *PIP1*;*1*, *PIP2*;*4* and *PIP2*;*5* transcripts compared with non-treated plants exposed to root hypoxia, but not under aerated conditions (Table S1; Fig. 3a,b).

## Immunodetection of PIPs

**Fig. 4:**
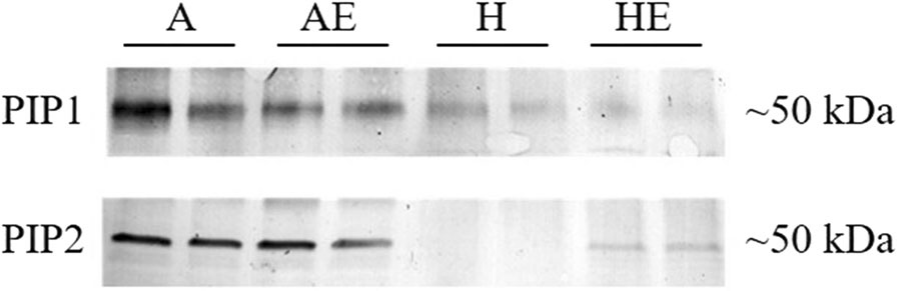
Western-blot analyses of plasma membrane intrinsic proteins (*PIP*s) in well-aerated trembling aspen (*P. tremuloides*) plants (A), well-aerated plants treated with ethylene (AE), plants exposed to hypoxia (H) and in plants subjected to hypoxia and ethylene treatment (HE) for one week. Results from two independent biological replicates of each treatment are shown in the figure. Antibodies against *Zea mays* PIP1 and PIP2 were used for the immunodetection. Full-length blots were presented in Supplementary Fig. S4

Antibodies against PIP1 and PIP2 were used for the immunodetection of PIPs following SDS PAGE. Immunoreactive bands at ~ 50 kDa were detected (Fig. 4; Fig. S4). The staining intensity of the immunoreactive bands with both antibodies was weaker in the roots of hypoxic plants compared with the well-aerated treatment. Under hypoxia, the immunoreactive bands that were detected with the anti-PIP2 antibody showed an increase in intensity in the roots treated with ethylene, but no effect of ethylene on PIP1 was detected (Fig. 4; Fig. S4).

## Discussion

The results demonstrate that the application of exogenous ethylene to roots partly alleviated the effects of hypoxia on root hydraulic conductance and leaf gas exchange. The effects of ethylene on *PIP* transcript profiles and protein abundance under hypoxic conditions were in parallel with changes of root hydraulic conductance and leaf transpiration rate, suggesting that the aquaporin-mediated water transport was the likely target of ethylene action.

Root hypoxia resulted in a drastic decrease of *K*_r_ in trembling aspen plants. A decrease of root water transport under O_2_ deficiency has been reported for different plant species [11]. Reduced water uptake under hypoxic conditions can be attributed to the impaired aquaporin-mediated water transport [8]. The O_2_ deficiency results in a transition from aerobic respiration to fermentation triggering energy crisis and causing cell acidosis which, in addition to reduced protein synthesis and phosphorylation, inhibits aquaporin gating [10, 11] and transmembrane water transport [34].

Similar to the earlier study [19], trembling aspen plants treated with exogenous ethylene under root hypoxia showed significantly higher *K*_r_ compared with plants that did not receive the ethylene treatment. In the present study, the effects of root hypoxia and ethylene on *K*_r_ were associated with the changes in aquaporin abundance. The relative transcript abundance of several *PIP*s responded differently to hypoxia and ethylene treatments. As limited information about aquaporins in *Populus tremuloides*, only several *PIP1*s and *PIP2*s were studied in our study. Both *PIP1*;*2* and *PIP2*;*2* transcripts were down-regulated while *PIP2*;*1* and *PIP2*;*5* transcripts were slightly up-regulated by hypoxia. The gene expression of *PIP1*;*1*, *PIP2*;*4*, *PIP2*;*5* were not markedly altered by the hypoxia treatment, but were significantly upregulated by the ethylene treatment under hypoxia. Western blot analyses confirmed that hypoxia decreased the overall protein abundance of both PIP1s and PIP2s, but ethylene-treated plants showed higher PIP2s expression than the plants receiving no exogenous ethylene. In a previous study, *PIP2*;*4* displayed striking up-regulation following defoliation accompanied by a recovery of *L*_pr_ in *P. tremuloides* demonstrating that this aquaporin plays an important role in regulation of water transport [35]. It should also be noted that *PIP2*;*5* has been demonstrated to be the major root aquaporin in poplars *P. tremula*×*tremuloides* [36] and *P. trichocarpa*×*deltoides* [37], with a high permeability coefficient when expressed in *Xenopus* oocytes, pointing to the functional significance of its enhanced expression by ethylene in our study.

Although ethylene treatments increased the expression abundance of *PIP1*;*1*, *PIP2*;*4 and PIP2*;*5*, the protein immunodetection results only showed an overall higher intensity of PIP2s under hypoxia, suggesting that PIP2s are likely the main targets of ethylene. Earlier studies demonstrated that ethylene induced the expression of *PIP2;1* in *Hevea brasiliensis* [28] and inhibited the *PIP2;1* expression in *Rosa hybrida* [32, 38]. Ethylene can also affect the post-transcriptional regulation of plant aquaporins. It was reported that ethylene increased the C-terminal phosphorylation of *Arabidopsis* PIP2;1 protein and promoted cell water transport rate [29]. Given that aquaporins regulate root water transport [39] and that PIP2s are the main aquaporins involved in water transport [40], the increase in the abundance of PIP2s might play an important role in the amelioration of root hydraulics by ethylene. However, the possibility that the increase in the abundance of PIP2s may also affect the transport of other molecules that alter responses to hypoxia should not be excluded since some of the aquaporins are also involved in the transport of lactic acid [23], O_2_ [15] and CO_2_ [24].

In our study, root hypoxia led to the upregulation of *ERF*s transcript abundance in hypoxic roots, indicating a likely enhancement of ethylene levels in roots under hypoxic conditions [41]. However, we observed no change in the transcript abundance of genes related to endogenous ethylene synthesis, including *ACS1*, *ACS2* and *ACO1*, by both hypoxia and exogenous ethylene (Fig. S5). It was reported that the entrapment of ethylene in the tissues of plants by stagnant water is the main factor responsible for its accumulation in roots [30], which may partly explain upregulation of *ERF*s by hypoxia even in the absence of increased ethylene synthesis. Under hypoxia, exogenous ethylene treatments increased *ERF18*, *ERF35* and *ERF71* transcript abundance compared to non-ethylene treatments, suggesting that exogenous ethylene treatment caused accelerated ethylene accumulation in trembling aspen roots. Thus, compared to non-ethylene treated plants, the increased ethylene accumulation in roots with exogenous ethylene treatments may contribute to the acclimation of plants to hypoxia by increasing the transcript abundance of PIP2 aquaporins and enhancing root hydraulics. In contrast, ethylene treatment did not change the expression levels of *ERF*s under aerated conditions, possibly due to the expulsion of dissolved ethylene by the air pumps in the hydroponic culture. On the other hand, the biosynthesis of ethylene in plants requires free O_2_ as a substrate [28] which may explain the observed interactions between hypoxia and ethylene treatments in *ERF35* and *ERF71* levels.

In the present study, ethylene treatment enhanced the gas exchange parameters in trembling aspen seedlings under hypoxic conditions. Photosynthetic responses to root hypoxia differ between plant species and are often used as indicators of hypoxia tolerance. In hypoxia-sensitive maize, net photosynthesis gradually decreased in response to flooding [29], but was maintained nearly unchanged in hypoxia-tolerant species *Melaleuca cajuputi* [42]. Other than the formation of adventitious roots and aerenchyma tissues to increase O_2_ supply to roots, the tolerance mechanisms that are responsible for this genetic diversity are still little understood [43]. In the present study, *P*_n_, *g*_s_ and *T*_r_ of trembling aspen showed sharp decreases in response to root hypoxia. However, ethylene treatments significantly increased *P*_n_, *g*_s_ and *T*_r_ under hypoxia and root porosity remained unchanged. It is well acknowledged that root hydraulics play a major role in restricting water delivery to leaves and are tightly linked to gas exchange [35, 44]. Aquaporins are essential in the adjustment and synchronization of root and leaf hydraulics. As demonstrated in partly defoliated trembling aspen, changes of root hydraulic conductivity and leaf transpiration rates were accompanied by major dynamic changes of the PIP expression in roots and leaves at different times and different degrees of defoliation [35]. In our study, since root hydraulic conductance was enhanced by the ethylene treatment in hypoxic plants, it is reasonable to assume that the amelioration of aquaporin-mediated root hydraulics through changes in the expression of PIPs was a likely contributor to the increase of gas exchange. Additionally, hypoxic treatment reduced *P*_m_ in trembling aspen which were similar with previous study with flooded *Genipa americana* seedlings [45]. However, ethylene treatment markedly increased *I*_m_ in hypoxic plants, without affecting *P*_m_. The change of light-saturated point in plants has been often reported to be due to the electron transport saturation and is reflected by the changes in chlorophyll fluorescence parameters [45]. In our study, no effects of ethylene on chlorophyll fluorescence were detected (data no shown). Furthermore, hypoxiatreatment also greatly hampered the *g*_s_ responses to light. Taken together, the results point to stomatal constrains due to reduced water delivery to leaves as the major factor contributing to the photosynthetic responses of hypoxic plants to ethylene.

## Conclusions

In conclusion, our study demonstrated that the treatment with exogenous ethylene altered aquaporin abundance, root hydraulics, and leaf gas exchange under root hypoxia conditions. The results point to the aquaporin gene expression, including *PIP2;4* and *PIP2;5*, is among the targets of ethylene action in trembling aspen under hypoxia conditions, which enhances the aquaporin-mediated root water transport and, consequently, leaf gas exchange.

## Methods

### Growth conditions and hypoxia treatment

Trembling aspen (*Populus tremuloides* Michx.) seeds were collected and identified by Dr. Wenqing Zhang (Department of Renewable Resources, University of Alberta) from the North Saskatchewan River valley (Edmonton, AB, Canada). Authorization is not required for seed collection in this area. Seeds were sterilized twice with 1 % (v/v) sodium hypochlorite and germinated in horticultural soil in a controlled growth room with 18 h photoperiod, 22/18^o^C (day/night) temperature, 400 µmol m^− 2^ s^− 1^ photosynthetic photon flux density and 50 % relative humidity. When five-weeks old, the plants were removed from pots and after rinsing the roots free of soil, they were transferred to 40 L polyethylene containers (60 × 40 × 20 cm) filled with a quarter-strength aerated modified Hoagland’s solution [46]. The plants were kept there for one week to recover from transplanting shock and then 24 plants were transferred into individual 1 L amber plastic flasks. Of these plants, 12 were randomly selected and subjected to aeration treatment by flushing the solution in the flasks with air pumps to maintain a dissolved O_2_ concentration of about 7.5 mg L^− 1^ throughout the experiment. The remaining 12 plants were subjected to hypoxia treatment by flushing N_2_ gas (Praxair, Danbury, CT, USA) for 30 min through the solution until reaching an O_2_ level of 2 mg L^− 1^. When the desired O_2_ concentration was reached, the plants where left in the stagnant solution for one week. The dissolved O_2_ concentration, which was monitored during the experiment with the YSI-5000 O_2_ electrode (YSI Incorporated, Yellow Springs, OH, USA) and declined in the stagnant solution to less than 1 mg L^− 1^ over the one-week treatment period (Fig. S6). All plant measurements and sample collection were carried out on the seventh day of treatments.

### Ethylene treatment

Half of the plants in the aerated control and hypoxia treatments (*n* = 6, respectively) were treated with exogenous ethylene at the beginning of the treatments. Ethylene was supplied to the hydroponic solution from the compressed gas cylinder through a tube stretched to the bottom of plastic bottles [19]. Dissolved ethylene concentration was determined at the onset of ethylene treatment with the headspace analysis method [47]. Briefly, 5 ml sample of hydroponic solution was collected into a gas-tight vacutainer. The headspace (10 %) was prepared by replacing water with N_2_, and the vacutainers were shaken at 1,400 RPM for 5 min. From the headspace, 100 µl gas samples were collected and injected into the Varian 430-GC gas chromatograph (Varian, Palo Alto, CA, USA) equipped with a HP Plot-Q column (30 m×0.53 mm×40 μm, Agilent, Santa Clara, CA, USA). The concentration of dissolved ethylene was calculated based on the Henry’s Law and a constant of 4.9 × 10^− 3^ M atm^− 1^ was used [47, 48]. The ethylene treatment resulted in an average dissolved ethylene concentration of 3.85 mmol L^− 1^.

### Leaf gas exchange

One fully extended leaf was randomly selected from each of the six plants per treatment during 9:00 to 12:00 h to measure net photosynthesis rate (*P*_n_), stomatal conductance (*g*_s_) and transpiration rate (*T*_r_) using a LI-6400XT Portable Photosynthesis System with a 2 × 3 cm^2^ red-blue light chamber (Li-Cor, Lincoln, NE, USA). The reference CO_2_ concentration was set to 400 µmol mol^− 1^; the flow rate was 200 µmol s^− 1^. The leaf chamber temperature was maintained at 20 °C, and PPFD was 400 µmol m^− 2^ s^− 1^ of the red-blue light spectrum. To evaluate the effects of treatments on leaf gas exchange under varying light conditions, light responses of *P*_n_ and *g*_s_ were established by setting an automated program with a starting photosynthetic photon flux density (PPFD) of 1500 µmol m^− 2^ s^− 1^, followed by 1200, 1000, 800, 500, 300, 200, 100, 50, 20 and 0 µmol m^− 2^ s^− 1^. Parameters were auto-logged when *P*_n_ reached steady state. Light saturated *P*_n_ (*P*_m_) and light saturation point (*I*_m_) were estimated by fitting the data to a modified rectangular hyperbole model [49].

### Root hydraulic conductance

Root hydraulic conductance (*K*_r_) was measured with a high-pressure flow meter (HPFM, Dynamax Incorporated, Houston, TX, USA). During the measurements of six plants in each treatment, roots were kept in the respective treatment solutions. Linear regression between the supplied pressure and flow rate was obtained by applying increasing pressure to the roots, and the slope was calculated as *K*_r_ [50].

### mRNA expression profiling

After one week of treatment, primary roots (3–5 cm-long distal root segments) of four to six plants from each treatment were collected and kept in liquid nitrogen before being transferred to -80^o^C freezer. Samples were ground with a mortar and pestle in liquid nitrogen. Total RNA was extracted with a Plant RNeasy extraction kit (Qiagen, Valencia, CA, USA). First strand of cDNA was synthesized from 1 µg total RNA using a Reverse Transcription Kit (Qiagen). Quantitative RT-PCR was employed to analyze RNA expression using *JIP1* (AJ407583.1) and *ACTIN1* (Potri.001G309500) as reference genes, which did not change significantly across all tested samples (*P* = 0.36). The transcript abundance of *PIP1*;*1* (AJ849323.1), *PIP1*;*2* (AJ849322.1), *PIP2*;*1* (AJ849324.1), *PIP2*;*2* (AJ849325.1), *PIP2*;*3* (AJ849326.1), *PIP2*;*4* (AJ849327.1) and *PIP2*;*5* (AJ849328.1) were determined. These PIPs have been proven to be functional and stress responsive in poplar plants [35]. The transcript abundance of five ethylene responsive factors (ERFs), *ERF17*, *ERF18*, *ERF35*, *ERF71* and *ERF76*, was determined since these genes were reported to be induced by ethylene in *P. tremula* × *tremuloides* [41]. The relative transcript abundance was calculated using the 2^−∆Ct^ method. The primers for trembling aspen *PIP*s [35] and *ERF*s [41] were designed as reported in the corresponding references (Table S2).

### SDS-PAGE and immunoblotting

SDS gel electrophoresis was performed to separate proteins (10 µg) extracted from roots after one week of treatment. Gel was transferred to a PVDF membrane (Bio-Rad, Hercules, CA, USA) by electroblotting at 100 V for 1 h. The membrane was blocked in 1 % BSA in TBST buffer (20 mM Tris pH 7.5, 150 mM NaCl and 0.1 % Tween 20) for 3 h and then incubated with the anti-PIP1 (1:500) or -PIP2 (1:1000) primary antibody overnight at 4^o^C. The anti-PIP1 antibody (R-4445) was raised against an amino peptide from ZmPIP1;5 and the anti-PIP2 antibody (R-2493) was raised against an amino peptide from ZmPIP2;4 and was shown to recognize PIP1s and PIP2s in maize and *Larix laricina* (Provided by Dr. F. Chaumont) [51, 52]. The PIP antibodies were raised in rabbit against maize (*Zea mays*) PIPs. The membrane was rinsed 5 times for 5 min with TBST and then incubated in GAR IgG antibody (1:2000) (Sigma-Aldrich, Oakville, ON, Canada) for 1 h at room temperature. After rinsing the membrane with TBST 5 times for 5 min, alkaline phosphatase (Bio-Rad) was conjugated to detect signals.

### Statistical analysis

The means and standard errors of aerated plants (A), aerated plants treated with ethylene (AE), hypoxic plants (H), and hypoxic plants treated with ethylene (HE) were calculated for each of the measured parameters. Two-way ANOVA was used to examine the effects of aeration (aerated vs. hypoxia treatments) and ethylene treatments (with or without ethylene). The data that did not meet the ANOVA assumptions of normality of distribution and homogeneity of variance were transformed with a square-root function. Comparisons between different treatment means were conducted using LSD test (α = 0.05).

## Supplementary information

**Additional file 1: Table. S1**. Effects of hypoxia and ethylene on the parameters determined in trembling aspen (*P. tremuloides*). **Table S2.** List of qRT-PCR primers used in this study. **Figure S1**. Light response of net photosynthesis and stomatal conductance in trembling aspen (*P. tremuloides*). **Figure S2**. Root length and shoot length in trembling aspen (*P. tremuloides*). **Figure S3**. Root porosity in trembling aspen (*P. tremuloides*). **Figure S4**. Full length original Western-blot pictures of PIP1s (a) and PIP2s (b) in trembling aspen (*P. tremuloides*). **Figure S5**. Transcript abundance of endo-ethylene synthesis related genes (*ACO1*, *ACS1* and* ACS2*) in trembling aspen (*P. tremuloides*). **Figure S6**. Dissolved oxygen concentration during one-week treatments in trembling aspen (*P. tremuloides*).

## Data Availability

The datasets generated or analyzed during the current study are available from the corresponding author on reasonable request.

## Abbreviations

A: Aerated plants.
AE: Aerated plants treated with ethylene.
ERF: Ethylene response factor.
*g*_*s*_: Stomatal conductance.
H: Hypoxic plants.
HE: Hypoxic plants treated with ethylene.
*I*_m_: Light saturation point.
*K*_*r*_: Root hydraulic conductance.
PIP: Plasma membrane protein.
*P*_*m*_: Light saturated net photosynthesis rate.
*P*_*n*_: Net photosynthetic rate.
*T*_r_: Transpiration rate.
