# Supplementary material for: Ethylene enhances root water transport and aquaporin expression in trembling aspen (Populus tremuloides) exposed to root hypoxia
Source: BMC Plant Biol. 2021 May 21;21:227. doi: 10.1186/s12870-021-02995-7 (PMC8140438; doi:10.1186/s12870-021-02995-7)
Supplement: Supplementary file 1 — Additional file 1: Table. S1. Effects of hypoxia and ethylene on the parameters determined in trembling aspen (P. tremuloides). Table S2. List of qRT-PCR primers used in this study. Figure S1. Light response of net photosynthesis and stomatal conductance in trembling aspen (P. tremuloides). Figure S2. Root length and shoot length in trembling aspen (P. tremuloides). Figure S3. Root porosity in trembling aspen (P. tremuloides). Figure S4. Full length original Western-blot pictures of PIP1s (a) and PIP2s (b) in trembling aspen (P. tremuloides). Figure S5. Transcript abundance of endo-ethylene synthesis related genes (ACO1, ACS1 and ACS2) in trembling aspen (P. tremuloides). Figure S6. Dissolved oxygen concentration during one-week treatments in trembling aspen (P. tremuloides). [file 12870_2021_2995_MOESM1_ESM.docx]

**Table** **S1** Effects of hypoxia and ethylene on the parameters determined in trembling aspen (*P. tremuloides*) in this study. *P*-values of the two-way ANOVA are shown (*n* = 3–6). *P*_n_ net photosynthesis rate, *g*_s_ stomatal conductance, *T*_r_ transpiration rate, *P*_m_ estimated light-saturated photosynthesis, *I*_m_ light saturation point, *K*_r_ root hydraulic conductance

|  | Variable | *P*-value | | |
| --- | --- | --- | --- | --- |
|  |  | Hypoxia | Ethylene | Hypoxia × Ethylene |
| Physiological  Measurements | *P*_n_ | **<0.001^***^** | **0.008^**^** | 0.502 |
|  | *g*_s_ | **<0.001^***^** | 0.338 | 0.181 |
|  | *T*_r_ | **<0.001^***^** | **<0.001^***^** | 0.554 |
|  | *P*_m_ | **0.002^**^** | 0.45 | 0.231 |
|  | *I*_m_ | **<0.001^***^** | **0.017^*^** | **0.042^*^** |
|  | *K*_r_ | **<0.001^***^** | 0.344 | 0.107 |
| Relative Transcript Abundance | *ERF17* | **<0.001^***^** | 0.172 | 0.157 |
|  | *ERF18* | **0.001^**^** | 0.13 | 0.067 |
|  | *ERF35* | **<0.001^***^** | **<0.001^***^** | **0.003^**^** |
|  | *ERF71* | **<0.001^***^** | **<0.001^***^** | **<0.001^***^** |
|  | *ERF76* | **<0.001^***^** | 0.328 | 0.415 |
|  | *PIP1;1* | 0.372 | 0.074 | **0.018^*^** |
|  | *PIP1;2* | **0.022^*^** | 0.056 | 0.42 |
|  | *PIP2;1* | **0.016^*^** | 0.356 | 0.536 |
|  | *PIP2;2* | **0.003^**^** | 0.396 | 0.171 |
|  | *PIP2;3* | 0.876 | 0.633 | 0.069 |
|  | *PIP2;4* | 0.231 | **0.026^*^** | **0.043^*^** |
|  | *PIP2;5* | **0.002^**^** | **0.02^*^** | **0.025^*^** |

^*^ *P* ≤ 0.05

^**^ *P* ≤ 0.01

^***^ *P* ≤ 0.001

**Table S2** qRT-PCR primers for trembling aspen (*Populus tremuloides*) genes

| Gene |  | DNA sequence |
| --- | --- | --- |
| *PIP1;1* | Forward | 5’-CTGCAATCATCTTCAACAAGG-3’ |
|  | Reverse | 5’-GCTCTGATCACAACCTGTGG-3’ |
| *PIP1;2* | Forward | 5’-CGAGAATCACATCTCTCAACAAC-3’ |
|  | Reverse | 5’-ACCACAATACCAAATTCACATG-3’ |
| *PIP2;1* | Forward | 5’-GTAAGAGAGATCGGATTACGATGG-3’ |
|  | Reverse | 5’-CACGAGAAAACACAAAACTACCAC-3’ |
| *PIP2;2* | Forward | 5’-ACAGTTACCAAGCCGAGAGAGAC-3’ |
|  | Reverse | 5’-GATCCTTTCTCTGTAGTCGGTAC-3’ |
| *PIP2;3* | Forward | 5’-CAACGTTATGTCATGGTTGTGTG-3’ |
|  | Reverse | 5’-AAGACTAACACAGAGCACTTGC-3’ |
| *PIP2;4* | Forward | 5’-AGAGGGTCTGGGAATCATGG-3’ |
|  | Reverse | 5’-TGAAGTGAATTCTAAGGCAAGC-3’ |
| *PIP2;5* | Forward | 5’-ATGCAAAGATTTAAGCAGTCC-3’ |
|  | Reverse | 5’-ATACACAGGGAAACCTTATGCAG-3’ |
| *ERF17* | Forward | 5’-CGAATACCAACATCAGGAAACAAA-3’ |
|  | Reverse | 5’-CCAGCAGCTCCTCCAACAA-3’ |
| *ERF18* | Forward | 5’-CAGGATCCAAGCTCTGCCA-3’ |
|  | Reverse | 5’-CCAGCAGCTCCTCCAACAA-3’ |
| *ERF35* | Forward | 5’-ACCTGACCAGATGGCTGCT-3’ |
|  | Reverse | 5’-TGACGTCGATACTGATGATGTGT-3’ |
| *ERF71* | Forward | 5’-AACCTCTTTAATCAGCAACCTTTTG-3’ |
|  | Reverse | 5’-CCTTCCCATGTTCCTGTTGA-3’ |
| *ERF76* | Forward | 5’-CTTCTCCCGCTTCTCCATCT-3’ |
|  | Reverse | 5’-CGAATCGCCCAGTGAACTT-3’ |
| *Jip1* | Forward | 5’-CTGGGAATGATCATGTGTTAATG-3’ |
|  | Reverse | 5’-ATACACAGGGAAACCTTATGCAG-3’ |
| *ACTIN1* | Forward | 5’-CGATGCCGAGGATATTCAAC-3’ |
|  | Reverse | 5’-ACCAGTGTGTCTTGGTCTACCC-3’ |
| *PtACO1* | Forward | 5’-GCGATCAGATTGAGGTCCTTAG-3’ |
|  | Reverse | 5’-GGTGGCCTTAAGTGATGGATTA-3’ |
| *PtACS1* | Forward | 5’-GGTGGCCTTAAGTGATGGATTA-3’ |
|  | Reverse | 5’-TCTCCGCTACGCTAATGAAATC-3’ |
| *PtACS2* | Forward | 5’-CCCTGGACAGAGAGACACTAAA-3’ |
|  | Reverse | 5’-CCTCTTCGACAACCTCAGAAATG-3’ |


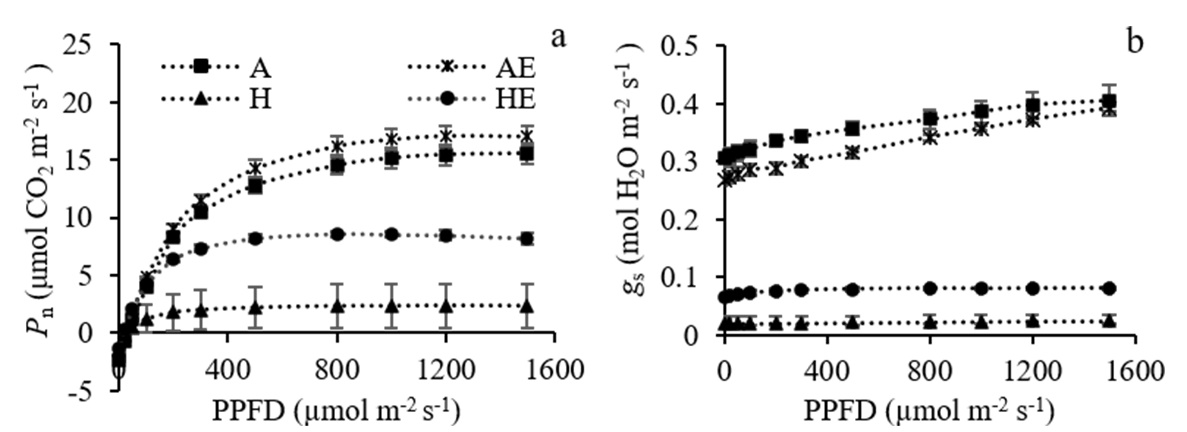


**Fig. S1** Light response of net photosynthesis (*P*_n_, a) and stomatal conductance (*g*_s_, b) in well-aerated trembling aspen (*P. tremuloides*) plants (A), well-aerated plants treated with ethylene (AE), plants exposed to hypoxia (H) and subjected to hypoxia and ethylene treatment (HE) for one week. Means ± SE (*n* = 3) are shown.


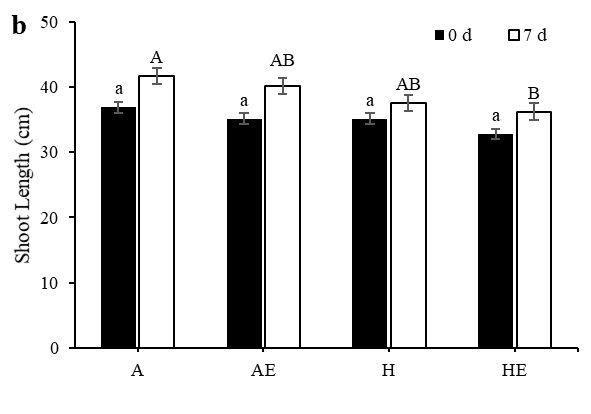

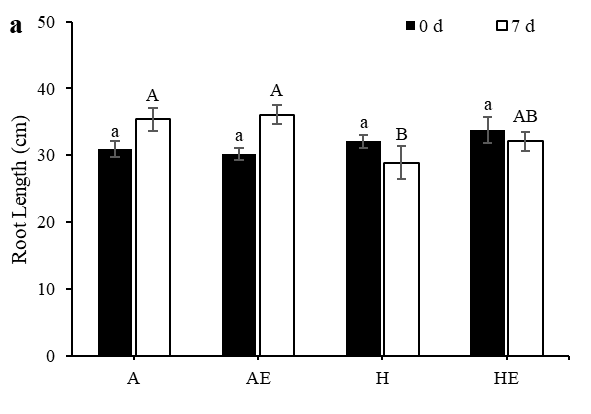


**Fig. S2** Root length (a) and shoot length (b) in well-aerated trembling aspen (*P. tremuloides*) plants (A), well-aerated plants treated with ethylene (AE), plants exposed to hypoxia (H) and subjected to hypoxia and ethylene treatment (HE) for one week. Means ± SE (*n* = 6) are shown. Different letters indicate significant difference (*P* ≤ 0.05).


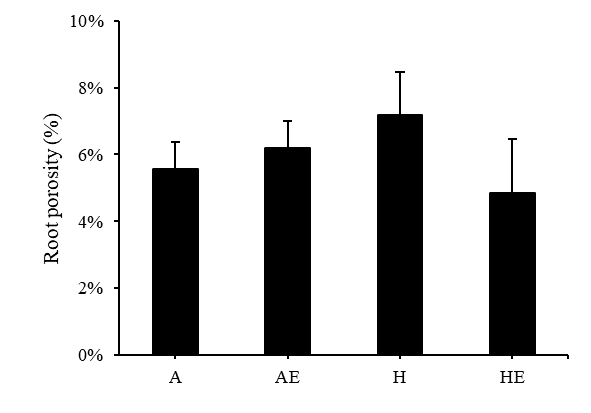


**Fig. S3**. Root porosity in well-aerated trembling aspen (*P. tremuloides*) plants (A), well-aerated plants treated with ethylene (AE), plants exposed to hypoxia (H) and subjected to hypoxia and ethylene treatment (HE) for one week. Means ± SE (n = 5) are shown.


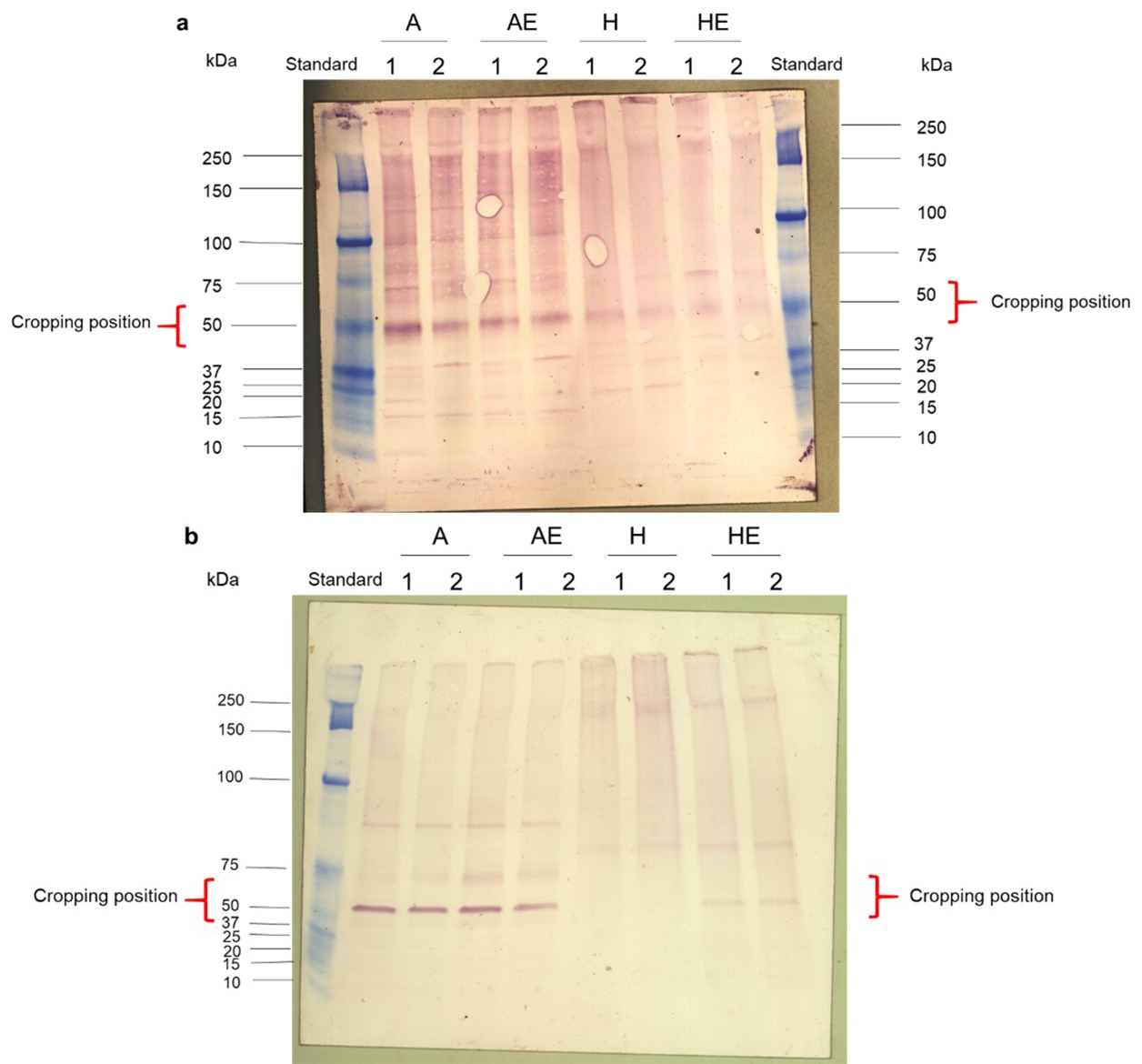


**Fig. S4** Full length original Western-blot pictures of PIP1s (a) and PIP2s (b) in well-aerated trembling aspen (*P. tremuloides*) plants (A), well-aerated plants treated with ethylene (AE), plants exposed to hypoxia (H) and in plants subjected to hypoxia and ethylene treatment (HE) for one week. Results from two independent biological replicates of each treatment were shown in the figure. Precision Plus Protein All Blue Prestained Protein Standards (Bio-Rad) was used as protein standard. Adobe Photoshop CC 2018 was used to change the pictures into black and white mode in the main text. Microsoft PowerPoint 2013 was used to add text. Bands at around 50 kDa were chosen to be presented in the main text, and other bands may represent oligometric forms of aquaporin proteins.

**Fig. S5** Transcript abundance of endo-ethylene synthesis related genes (*ACO1*, *ACS1* and *ACS2*) in well-aerated trembling aspen (*P. tremuloides*) plants (A), well-aerated plants treated with ethylene (AE), plants exposed to hypoxia (H) and subjected to hypoxia and ethylene treatment (HE) for one week. Means ± SE (*n* = 6) are shown. No significance was detected in one-way ANOVA (*ACO1*: *P* = 0.897; *ACS1*: *P* = 0.495; *ACS2*: *P* = 0.464).


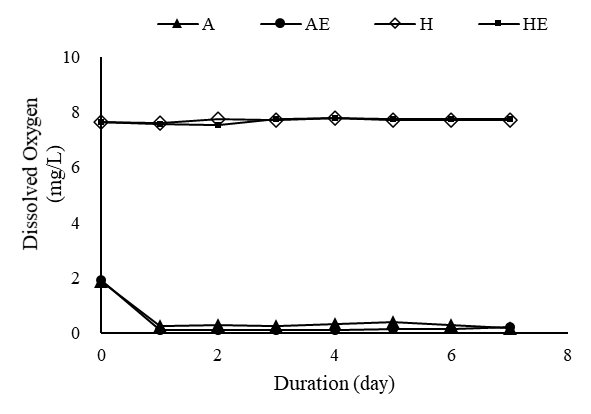


**Fig. S6** Dissolved oxygen concentration during one-week treatments in well-aerated trembling aspen (*P. tremuloides*) plants (A), well-aerated plants treated with ethylene (AE), plants exposed to hypoxia (H) and subjected to hypoxia and ethylene treatment (HE) for one week.
